# A Novel Sulfonated Derivative of β-Cyclodextrin Effectively Inhibits Influenza A Virus Infection in vitro and in vivo

**DOI:** 10.32607/20758251-2019-11-3-20-30

**Published:** 2019

**Authors:** E. P. Goncharova, Y. A. Kostyro, A. V. Ivanov, M. A. Zenkova

**Affiliations:** Institute of Chemical Biology and Fundamental Medicine, Siberian Branch of Russian Academy of Sciences, Lavrentiev Ave. 8, Novosibirsk, 630090, Russia; A.E. Favorsky Irkutsk Institute of Chemistry, Siberian Branch of Russian Academy of Sciences, Lermontov Str. 134, Irkutsk, 664033, Russia

**Keywords:** influenza virus, antiviral activity, sulfonated derivative of β-cyclodextrin

## Abstract

The development of novel drugs against the influenza virus with high efficiency and low toxicity is an urgent and important task. Previous reports have demonstrated that compounds based on sulfo derivatives of oligo- and polysaccharides possess high antiviral activity. In this study, we have examined the ability of a novel sulfonated derivative of β-cyclodextrin (KS-6469) to inhibit the influenza virus A/WSN/33 (H1N1) infection in vitro and in vivo. The antiviral potential of KS-6469 against the influenza virus was evaluated in Madin-Darby Canine Kidney epithelial cells treated with serially diluted KS-6469. We found out that KS-6469 completely inhibited viral reproduction after treatment of the infected cells with the compound for 48 h. Our data show that double intranasal treatment of mice with KS-6469 fully protected the animals from a lethal infection and significantly decreased the viral titers in the lungs of the infected animals. Thus, the novel sulfonated β-cyclodextrin derivative KS-6469 is a promising candidate for the development of antiviral drugs for preventing and treating the influenza infection.

## INTRODUCTION

The influenza virus causes respiratory diseases that are responsible for the death of up to 650,000 people each year worldwide [1]. The emergence of new drugresistant strains of influenza and the limited effectiveness of existing vaccines mean that the development of new, more effective antiviral compounds is critical in order to fight the virus. To this end, sulfonated polysaccharides are among the most promising antiviral compounds. This group of compounds includes sulfo derivatives of oligo- and polysaccharides containing O-sulfate and/or N-sulfamate moieties. The antiviral properties of sulfonated polysaccharides have been known for a long time. Ginsberg et al. investigated the antiviral properties of the capsular polysaccharide of Klebsiella pneumoniae and showed that this polysaccharide effectively suppresses the replication of the mumps virus [2]. It has previously been reported that sulfonated polysaccharides, including β-cyclodextrin, display antiviral activity against a number of enveloped viruses [3-7]. There is evidence that a number of sulfonated polysaccharides– in particular, sulfo derivatives of oligo- and polysaccharides containing O-sulfate groups–possess high antiviral activity [8-11]. Synthetic polymers of the N-sulfonate derivatives of poly(allylamine hydrochloride) have been shown to effectively inhibit the influenza A virus in vitro and ex vivo, mainly in the  late stages of the infection [12]. It is noteworthy that i-carrageenan, a sulfated polysaccharide, has been clinically tested, authorized for release, and sold in some countries for intranasal treatment of an influenza infection [13]. However, no data on the antiviral activity of the sulfo derivatives of carbohydrates containing sulfonate groups (C-sulfates) are available.

In this study, we investigated the antiviral properties of a sulfonated (C-sulfate) oligosaccharide (a bisulfite derivative of oxidized β-cyclodextrin KS-6469) against the influenza A/WSN/33 (H1N1) (IAV) virus in vitro and in vivo. The results revealed that KS-6469 effectively inhibits the replication of IAV in MDCK cells by acting in the late stages of the viral infection, and that it possesses virucidal properties. The results obtained on a mouse model of lethal influenza infection in vivo confirmed the high antiviral potential of this compound.

## EXPERIMENTAL

**Synthesis of the KS-6469 compound**

The KS-6469 compound was developed and synthesized at the A.E. Favorsky Irkutsk Institute of Chemistry (Siberian Branch of the Russian Academy of Sciences) based on commercially available β-cyclodextrin (KLEPTOSE®, Belgium). A detailed description of the synthetic procedure and the physicochemical properties of KS-6469 will be published separately. Oseltamivir (Tamiflu®, Switzerland) was prepared as a solution in phosphate-buffered saline (PBS) for oral gavage administration.

**Cytotoxicity of KS-6469 in MDCK cells**

*The MTT assay*. The cytotoxicity of KS-6469 was evaluated using the 3-(4,5-dimethylthiazol-2-yl)-2,5-diphenyltetrazolium bromide (MTT) test as described previously [14]. Madin-Darby Canine Kidney (MDCK) cells were received from the Bank of Cell Cultures (Institute of Cytology, St. Petersburg, Russia). The cells were maintained in Iscove’s Modified Dulbecco’s Medium (IMDM; Sigma, USA) supplemented with 5% fetal bovine serum (Sigma, USA), 100 units/L penicillin, 100 mg/mL streptomycin, and 0.25 mg/mL amphotericin (antibiotic-antimycotic solution, Sigma, USA) at 37°C in a humidified atmosphere containing 5% CO_2_ (from here on referred to as “standard conditions”). Briefly, MDCK cells were seeded into 96-well plates and grown to confluence under standard conditions. The medium was replaced with a fresh medium containing serially diluted KS-6469, and the cells were incubated for another 24 h under standard conditions. Aliquots of the MTT solution were added to each well, and incubation was continued for an additional 3 h. The dark blue formazan crystals formed within live cells were solubilized with dimethyl sulfoxide (DMSO); absorbance was measured at 570 nm in a MultiskanTM FC plate reader (Thermo LabSystems, Finland). The IC_50_ was determined as the compound concentration required to decrease the A_570_ to 50% of the control (DMSO alone) and was determined by interpolation from the dose-response curves.

*Real-time cell analysis.* The cytotoxic effects of KS-6469 on the MDCK cells were measured on an xCELLigence real-time cell analyzer (ACEA Biosciences Inc., USA), which is based on the microelectronic biosensor technology. The electrode impedance displayed as the cell index (CI) value was measured to compare the status of treated and untreated cells. The cells were plated in 16-well plates (ACEA Bioscience Inc., USA) in IMDM supplemented with 10% FBS and a antibiotic-antimycotic solution and incubated overnight (20 h) under standard conditions. The growth medium was then removed, the cells were washed with PBS, and 150 μL of IMDM either containing different concentrations of KS-6469 or without the compound was added to each well. The cells were then incubated under standard conditions for 65 h with cell viability monitored every 15 min using the xCELLigence real-time cell analyzer. The points were run in duplicate, and the IC_50_ value was calculated using the xCELLigence real-time cell analyzer software. The concentration of KS-6469 at which the diagram of CI for the treated cells coincided with that of CI for the untreated cells was defined as the maximum tolerated concentration (MTC).

**Determination of the antiviral activity of KS-6469 in MDCK cells**

The influenza virus strain A/WSN/33 (H1N1) was obtained from the Ivanovsky Institute of Virology (Moscow, Russia). MDCK cells were grown to confluence in 24-well plates under standard conditions. The cells were then infected with IAV at MOI 0.1 in a medium supplemented with KS-6469 (70–600 μg/mL) and incubated at 37°C for 24 or 48 h under standard conditions. Twenty-four or 48 h post-infection (p.i.), the cells were subjected to one freeze/thaw cycle (-20/20°C) and then the viral titer was determined by FFA as described previously [15]. The index of virus yield reduction (KI, %) and the chemotherapeutic index (CTI) were used as basic criteria for evaluating the efficacy of KS-6469 in vitro. The index of virus yield reduction was determined as:

KI = (T_c_ – T_o_)/T_c_ × 100%,

where T_c_ is the viral titer in the medium without KS- 6469, and T_o_ is the viral titer in the medium supplemented with KS-6469 [16]. CTI of the compound was defined as the ratio:

CTI = MTC / MEC,

where MTC is the maximum tolerated concentration, and MEC is the minimum concentration of the compound producing a 100-fold reduction in virus titer [17].

**Time-of-addition assay**

To determine the steps of the IAV life cycle that proved sensitive to the KS-6469 treatment, the MDCK cells were grown to confluence in 24-well plates and infected with IAV (MOI 0.1) for 1 h under standard conditions. The KS-6469 compound was added at a concentration of 5 mg/mL before, during, or after the IAV infection. After each period of incubation with the virus, the cells were washed with PBS and incubated with a fresh infection medium at 37°C. Forty-eight h p.i., the cells were subjected to one freeze/thaw cycle and the viral titer was determined by FFA.

**The NA-Fluor™ Influenza Neuraminidase Assay**

Neuraminidase (NA) activity was measured using the NA-Fluor™ Influenza Neuraminidase Assay kit (Applied Biosystems, USA), according to the manufacturer’s protocol. The assay is based on the NA enzyme cleaving the 2’-(4-methylumbelliferyl)-α- D-N-acetylneuraminic acid (MUNANA) substrate to release the fluorescent product, 4-methylumbelliferone. The fluorescent signal was measured using a CLARIOstar® fluorescence plate reader (BMG LABTECH, Germany). The assays were performed in triplicate.

**Hemagglutination assay**

The antiviral activity of KS-64649 was estimated using hemagglutination assay (HA). Viral suspensions were incubated with an equal volume of the medium with or without KS-6469. In this assay, a 0.5% suspension of chicken erythrocytes was used. The viral titer of the sample was calculated as the inverse value of the dilution at which the last agglutinated appearance was detected.

**Analysis of the cholesterol level in the viral envelope**

The cholesterol level in the viral envelopes was determined using the Amplex Red Cholesterol assay kit (Molecular Probes, USA), according to the manufacturer’s instructions. Briefly, the viruses were incubated with different concentrations of KS-646 for 6 h at 37°C then pelleted and re-suspended in Amplex Red reaction buffer. Methyl-β-cyclodextrin was used as a positive control. Fluorescence was then analyzed on a CLARIOstar® plate reader at an excitation wavelength of 550 nm and an emission wavelength of 590 nm. The assays were performed in triplicate.

**Virucidal activity of KS-6469**

Viral suspensions were incubated with an equal volume of the medium with or without KS-6469 for 3 or 6 h at 4, 20 or 37°C. The viral titer was estimated using the FFA. The assays were performed in triplicate.

**Enzyme-linked immunosorbent assay**

Enzyme-linked immunosorbent assays were performed using commercially available monoclonal antibodies against influenza virus hemagglutinin [IVC102], [1.B.408] and [B219M] (Abcam) as described previously [18].

**Antiviral activity of KS-6469 on the mouse model of lethal influenza infection**

***Animals.*** Female 4- to 6-week-old BALB/c mice were purchased from the State Research Center of Virology and Biotechnology Vector (Koltsovo, Russian Federation). The animals were kept in the vivarium of the Institute of Chemical Biology and Fundamental Medicine, SB RAS, with a natural light regime on a standard diet for laboratory animals (GOST [State Standard] R 5025892) in compliance with the international recommendations of the European Convention for the Protection of Vertebrate Animals used for Experimental and Other Scientific Purposes [19], as well as the rules of laboratory practice in the performance of pre-clinical studies in the Russian State Standards (R 51000.3-96 and 51000.4- 96). The experimental protocols were approved by the Inter-Institute Bioethics Commission of SB RAS (22.11 dated May 30, 2014).

***In vivo toxicity analysis.*** To evaluate the toxicity of KS-6469, the mice were treated by intraperitoneal or intranasal administration of varying amounts of the compound. Each of the experimental groups contained six BALB/c mice. The animals received KS-6469 in different doses intraperitoneally in 0.2 mL of PBS, or 250 mg/kg twice daily intranasally in 40 μL of PBS. The mice from the control groups received the same volume of PBS. After the treatment, signs of intoxication including general condition, weight, and depression of the central nervous system were assessed daily for 14 days.

Protective efficacy in mice. To test the protective efficacy of KS-6469, we evaluated weight changes, the survival rate, and viral titer in the lungs of the infected mice. BALB/c mice with an average weight of 14 to 16 g were divided into groups of six animals. The mice were anesthetized by intraperitoneal injection of tribromoethanol (Avertin®) and infected intranasally with 3 LD_50_ being equal to ~1.2 × 10^4^ ± 0.7 × 10^4^ FFU of IAV immediately after intranasal administration of KS-6469 at a dose of 250 mg/kg. The following day, the mice received a second dose of KS-6469 intranasally. The study included a positive control group that received oseltamivir daily (7.5 mg/ kg) for 5 days. The control mice received PBS instead of KS-6469. The animals were inspected daily and weighed for 14 days after infection. The degree of protection was estimated according to the reduction in the mortality rate. To assess the viral titer in the lungs, BALB/c mice were divided into groups of six animals. The mice were infected intranasally with 3 LD_50_ of IAV immediately after intranasal administration of KS-6469 at various doses. The following day, the mice received a second dose of KS-6469 intranasally. The control mice received PBS instead of KS-6469. The mice in each group were euthanized on day 3 p.i., and the lung tissue was harvested. The lungs were weighed and the medium was added at a ratio between the lung tissue and the medium of 1 : 10 (v/v). The homogenates were prepared using a Sonopuls HD 2070 ultrasonic homogenizer (Bandelin, Germany). The viral titers of the lung homogenates were determined using FFA.

**Statistical analysis**

## RESULTS

The data are expressed as the mean ± SD. The statistical analysis was performed using the two-tailed unpaired t-test. P-values of less than 0.05 were deemed statistically significant.

**Characterization of KS-6469**

The compound KS-6469 is a bisulfite derivative of oxidized β-cyclodextrin. The sulfonate moieties of bisulfite derivatives are bound directly to a carbon atom with the general formula (Gluox)7-Cx-(SO3Na)x, where (Gluox)7-Cx is oxidized β-cyclodextrin, x is the number of sulfonated carbon atoms, and SO3R is the bisulfite (sulfonate) group. The detailed structure and physicochemical properties of KS-6469 are the subject of ongoing research and will be published later.

**Cytotoxicity of KS-6469**

**Fig. 1:**
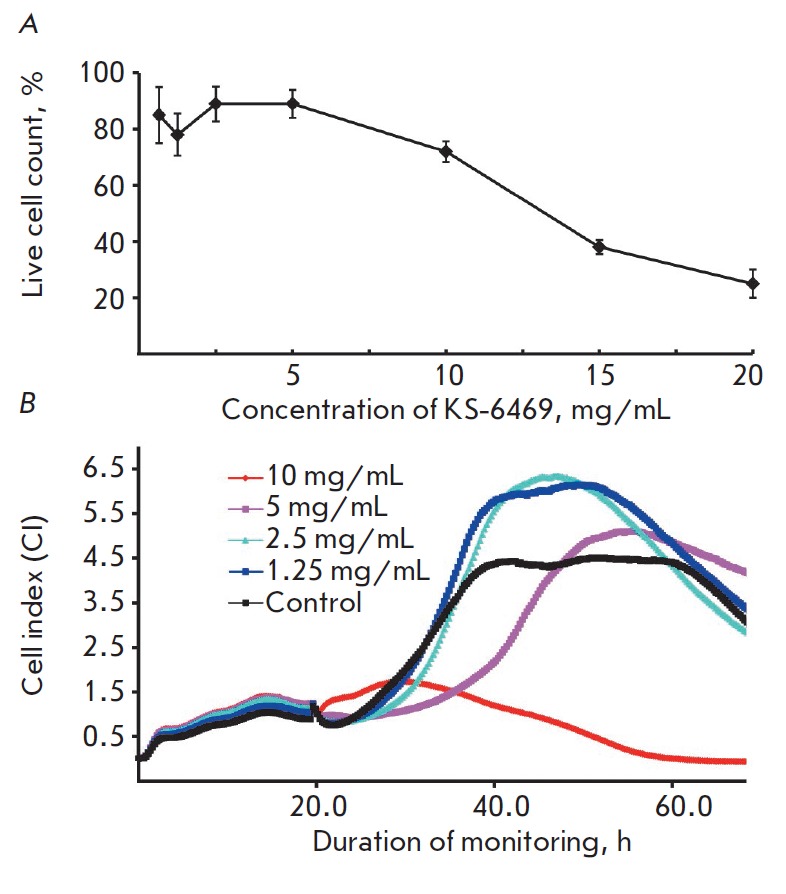
Cytotoxicity of KS-6469 with respect to MDCK cells. MDCK cells were exposed to different concentrations of KS-6469 and incubated at 37°C, followed by cell viability measurement by MTT assay (A); the viability of MDCK cells treated with KS-6469 was monitored by Real- Time Cell Analysis (B). Cells incubated in IMDM without KS-6469 were used as control

The cytotoxicity of KS-6469 toward the MDCK cells was examined. The half maximal inhibitory concentration (IC_50_) value (the concentration of KS-6469 that caused 50% cell lethality) was 15 ± 3.3 mg/mL, as determined by MTT assay after 24 h incubation with the compound (Fig. 1A). An analysis of the cytotoxic effect of KS-6469 on the cells following 65 h exposure to KS-6469 was measured using real-time cell analysis. The IC_50_ value obtained by this method was found to be 8.9 ± 1.3 mg/mL after 65 h exposure (Fig. 1B). These results confirmed the low cytotoxicity of KS-6469 toward eukaryotic cells, which reached 15 and 8.9 mg/mL for the 24 and 65 h exposure durations, respectively. Based on the data from these experiments, we can conclude that KS-6469 possesses low toxicity toward MDCK cells.

**Antiviral properties of KS-6469**

**Fig. 2:**
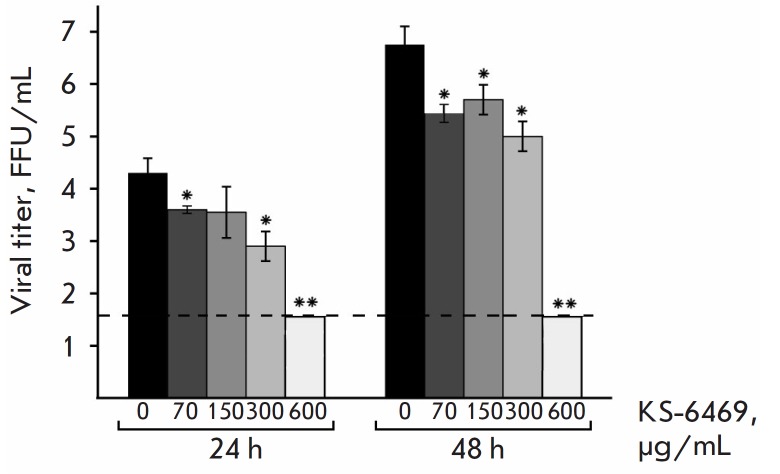
The replication of the influenza virus A/WSN/33 (H1N1) is effectively inhibited in MDCK cells by treatment with KS-6469. Various concentrations of KS-6469 were used to treat MDCK cells. The production of infectious viruses was determined by FFA 24 and 48 h p.i. The limit of detection for the FFA is 1.7 lg FFU/mL and is shown by a dotted line. Significance: *p < 0.05, **p < 0.01 vs. virus only (0)

The antiviral activity of KS-6469 was tested in vitro in the MDCK cells/IAV model of the influenza infection. The MDCK cells were simultaneously treated with different doses of KS-6469 and IAV (MOI 0.1). Virus quantification (Fig. 2) showed that replication was severely affected by KS-6469. Dosedependent inhibition of viral replication was observed at KS-6469 concentrations of 70–600 μg/mL. At 70 μg/mL KS-6469, the viral titer was significantly reduced: KI = 75% (60.0–80.2%) (p < 0.05). It should be noted that when the virus was incubated with 600 μg/mL KS-6469, viral replication was entirely suppressed by KS-6469 because the virus levels were undetectable in the culture medium at 48 h post-infection. Hence, the sulfonated derivative of β-cyclodextrin investigated in this study could significantly reduce the virus titer when used at a concentration > 70 μg/mL and entirely suppressed viral replication at 600 μg/mL.

**Fig. 3:**
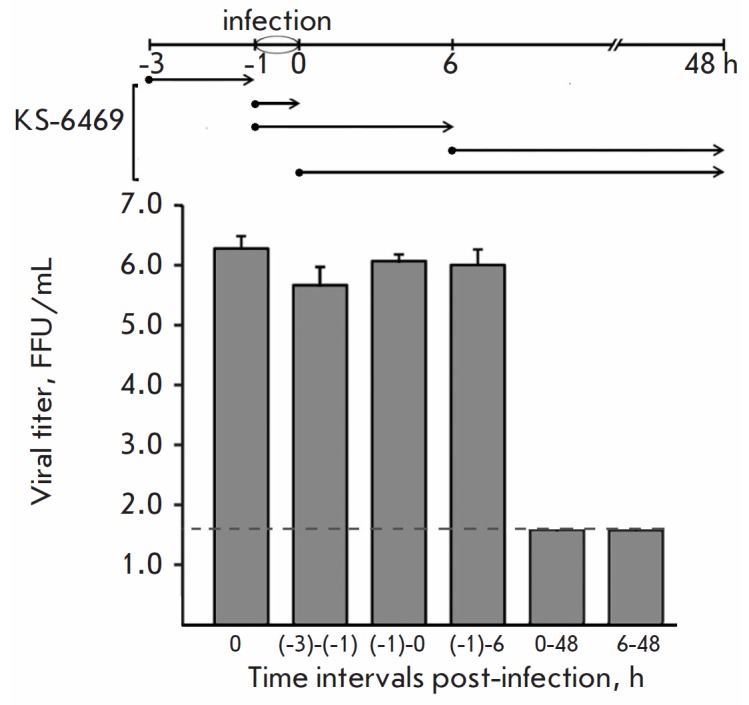
Time-of-addition experiment showing the mechanism of influenza virus inactivation by KS-6469. Cells were infected with IAV at MOI 0.1 for 1 h (from time point (-1) to 0 h); 5 mg/mL KS-6469 was then added to the cells at the indicated time points (•). After each incubation period (shown with arrows), the medium containing KS-6469 was replaced with a compound-free medium. At 48 h p.i., the supernatants were collected and the viral titers were evaluated by FFA. The data are presented as mean ±SD (n = 3). The limit of detection for FFA (1.7 lg FFU/mL) is shown with a dashed line

To gain insight into the mechanism of KS-6469 inhibition of IAV replication, we determined the step of the viral life cycle that was affected by the compound using time-of-addition assay. First, we investigated whether KS-6469 added to the cells prior to virus adsorption could protect the cells against infection. The cells were treated with KS-6469 2 h prior to IAV infection ((-3) to (-1) h) (Fig. 3). The viral yield showed no difference when compared with the virus-only control. Similarly, no antiviral activity was observed when KS-6469 was introduced into the well during infection ((-1) to 0 h) or during the early stages of viral replication ((–1 h) to 6 h p.i.). These results indicate that the compound directly affects neither the binding nor virus entry into the target cells, or its early release. Inhibition of viral replication was only observed either when KS-6469 was continuously present in the medium from the post-infection period (0 to 48 h) or when it was added 6 h after infection (Fig. 3).

**Effect of KS-6469 on the functional activity of influenza virus surface proteins.**

**Table 1:** HA titer of the virus after incubation with KS-6469

Compound	HA titer of the virus and incubation conditions			
	4°C		37°C	
	1 h	6 h	1 h	6 h
KS-6469, 5 mg/mL	64	32	16	4
PBS	64	64	64	64

Functional balance between influenza virus hemagglutinin (HA) and neuraminidase (NA) was found to be very important for successful viral replication and fitness. In order to determine whether KS-6469 affected the structure of viral hemagglutinin, we investigated virus attachment to erythrocytes. The influenza virus was treated with KS-6469 (5 mg/mL) for 1 or 6 h at 37 or 4°C, and the viral HA titer was evaluated using HA assay Table 1). Virus co-incubation with KS-6469 for 1 h at 37°C caused a fourfold reduction in the HA titer, and after 6 h of incubation HA was reduced 16-fold (Table 1). Interestingly, the HA titer did not change significantly after incubation for 6 h at 4°C in the presence of KS-6469 at the same concentration. The reduction in hemagglutinin binding suggests that KS-6469 could induce changes in the conformation of the surface epitopes of the protein.

**Fig. 4:**
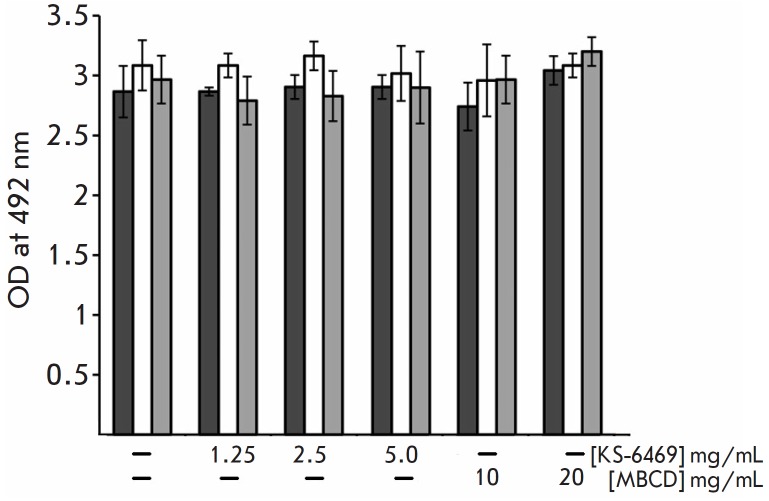
The effect of treatment with KS-6469 on the binding of virus hemagglutinin to specific monoclonal antibodies. The data are presented as mean OD ± SD (n = 3). OD is the optical density. The experiment was conducted in triplicate

The ability of KS-6469 to affect the structure of the surface epitope of viral hemagglutinin was analyzed using a set of monoclonal antibodies specific to hemagglutinin of IAV. The treatment of IAV with KS-6469 (5 mg/mL) at 37°C, which resulted in a 16-fold decrease in HA titer, changed neither the structure of virus hemagglutinin nor the efficacy of its binding to specific monoclonal antibodies (Fig. 4). It should be noted that the process of inhibiting HA of virus glycoproteins and, therefore, the infectivity of the virus particle, require a sufficiently long incubation with KS-6469 at a temperature of at least 37°C; so, we observed only a partial decrease in HA activity following incubation of the virus with the compound for 1 h.

**Fig. 5:**
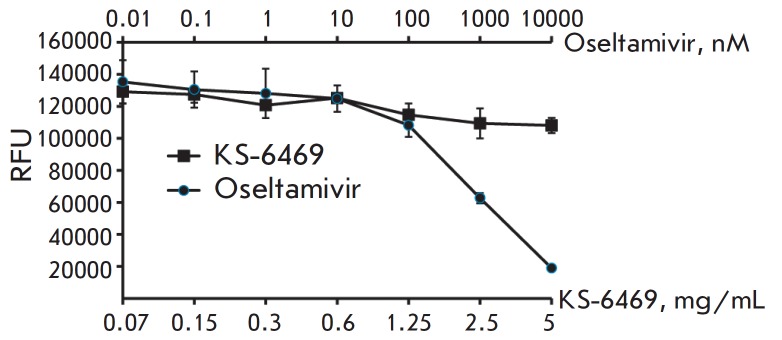
Neuraminidase activity assay. The influenza virus A/WSN/33 was incubated with KS-6469 or Oseltamivir for 6 h before addition of a MUNANA substrate and then incubated for 1 h at 37°C. The results are presented in RFU ± SD (n = 3). RFU stands for relative fluorescence units. The experiment was conducted in triplicate

**Fig. 6:**
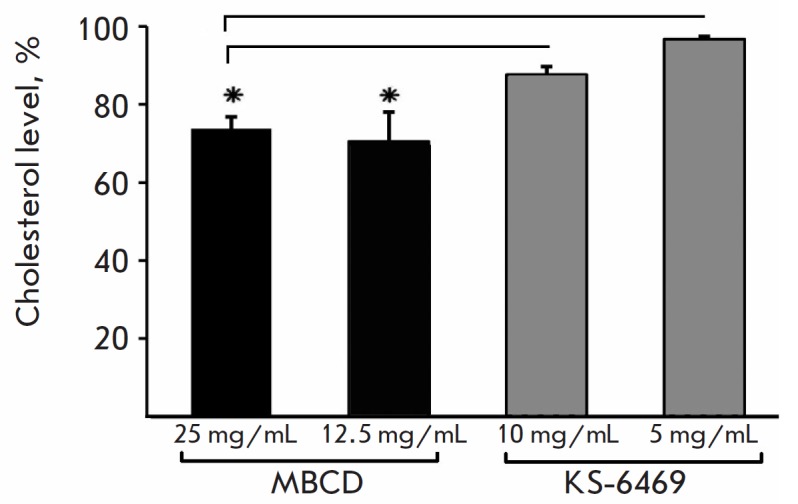
Cholesterol level in the IAV envelope following treatment with KS-6469 or MBCD. IAV was incubated with different concentrations of KS-6469 or MBCD for 6 h at 37°C, and cholesterol levels were detected using the Amplex Red Cholesterol assay kit (n = 3). The cholesterol level in the untreated virus was set as 100%. Significance: *p < 0.05. The experiment was conducted in triplicate

In order to gain an understanding of the mechanism by which K-6469 inhibits viral replication, we examined the inhibition of viral neuraminidase (NA), which mediates the release of viral progeny from infected cells and promotes viral transmission. The results (Fig. 5) indicated that KS-6469 (5 mg/mL) did not change the NA activity, suggesting that NA is not targeted by KS-6469. Given that the mechanism of the antiviral effect of another derivative of β-cyclodextrin, methyl β-cyclodextrin (MBCD), is associated with cholesterol removal from the viral envelope [20], we examined the cholesterol level of virus particles with and without KS-6469 or MBCD treatment. IAV was pretreated with various concentrations of KS-6469 and MBCD or left untreated (Fig. 6). A relative depletion of viral envelope cholesterol was observed after treatment with MBCD but not following treatment with KS-6469. Unlike MBCD, the antiviral effect of KS-6469 is, therefore, not associated with changes in the cholesterol content in the viral envelope. Taking into account these findings, a conclusion can be drawn that treatment with KS-6469 partially decreases the HA activity of the virus but does not change the NA activity and cholesterol content in the viral envelope.

**Virucidal properties of KS-6469**

It has been previously shown that sulfated polysaccharides possess virucidal activity [21, 22]. In the present study, a series of experiments was conducted to examine the ability of KS-6469 to directly inactivate influenza virus particles. The incubation of IAV with 5 or 2.5 mg/mL KS-6469 at 37°C for 3 h resulted in complete inactivation of virus infectivity. Treatment with 1.25 mg/mL KS-6469 followed by 3 h incubation at 37°C resulted in a ~2 lg reduction of viral titer, and no infectious virus particles were detected by FFA after 6-h incubation (Table 2). Incubation with 0.3 mg/mL KS-6469 at 37°C for 6 h did not affect viral infectivity. The lower incubation temperature resulted in significantly decreased virucidal properties for KS-6469. When virus particles were incubated with the compound at 4 or 20 °C, no effect on infectivity was observed. We hypothesized that the virucidal effect of KS-6469 might be mediated by the formation of aggregates of virus particles and the compound, and that a lower incubation temperature might drastically reduce the formation of such aggregates, thus diminishing the virucidal activity of the compound.

**Table 2:** Virus titer (lg FFU/mL) after treatment of the influenza virus with KS-6469

KS-6469 (mg/mL)	Incubation conditions					
	4°C		20°C		37°C	
	3 h	6 h	3 h	6 h	3 h	6 h
0.3	n.d.	5.4 ± 0.1	n.d.	4.9 ± 0.1	n.d.	4.8 ± 0.3
0.6	5.4 ± 0.1	4.9 ± 0.1	5.3 ± 0.1	4.9 ± 0.2	3.9 ± 0.1	1.9 ± 0.3
1.25	4.7 ± 0.3	5.1 ± 0.1	4.8 ± 0.2	4.6 ± 0.2	2.4 ± 0.2	< 1.7
2.5	5.0 ± 0.2	5.3 ± 0.3	4.5 ± 0.2	4.3 ± 0.2	< 1.7	< 1.7
5.0	n.d.	4.9 ± 0.4	n.d.	4.3 ± 0.4	< 1.7	< 1.7
PBS	5.1 ± 0.1	5.2 ± 0.2	5.1 ± 0.1	5.0 ± 0.3	4.4 ± 0.3	4.6 ± 0.1

n.d. – not determined.

**Antiviral activity of KS-6469 in the mouse model of influenza**

**Fig. 7:**
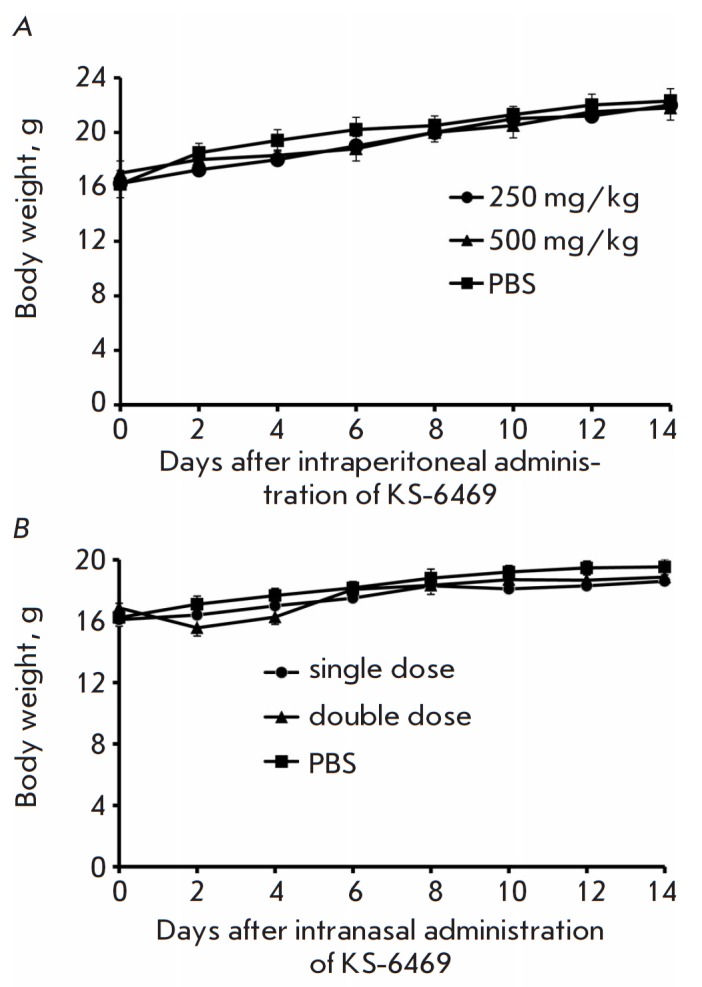
Effects of KS-6469 treatment on the body weight of healthy mice. BALB/c mice (n = 6) received KS-6469 at doses of 500 and 250 mg/kg once as an intraperitoneal injection in 200 μL of PBS (A), or once or twice intranasally at a dose of 250 mg/kg in 40 μL of PBS (B). Mean values ± SD are shown at each time point

***In vivo cytotoxicity of KS-6469.*** BALB/c mice were subjected to a single intraperitoneal injection of different doses of KS-6469 (250 mg/kg or 500 mg/kg) in PBS. The physical parameters and body weights of the animals were monitored daily (Fig. 7). A single injection of the compound at a maximum dose of 500 mg/kg did not cause any weight loss or changes in the general condition, or death, thus indicating that KS-6469 exhibits low toxicity (Fig. 7A). Mice treated twice with KS-6469 intranasally (dose 250 mg/kg) had minimal weight loss and no signs of toxic side effects during the entire observation period (Figure 7A and Figure 7B).

**Fig. 8:**
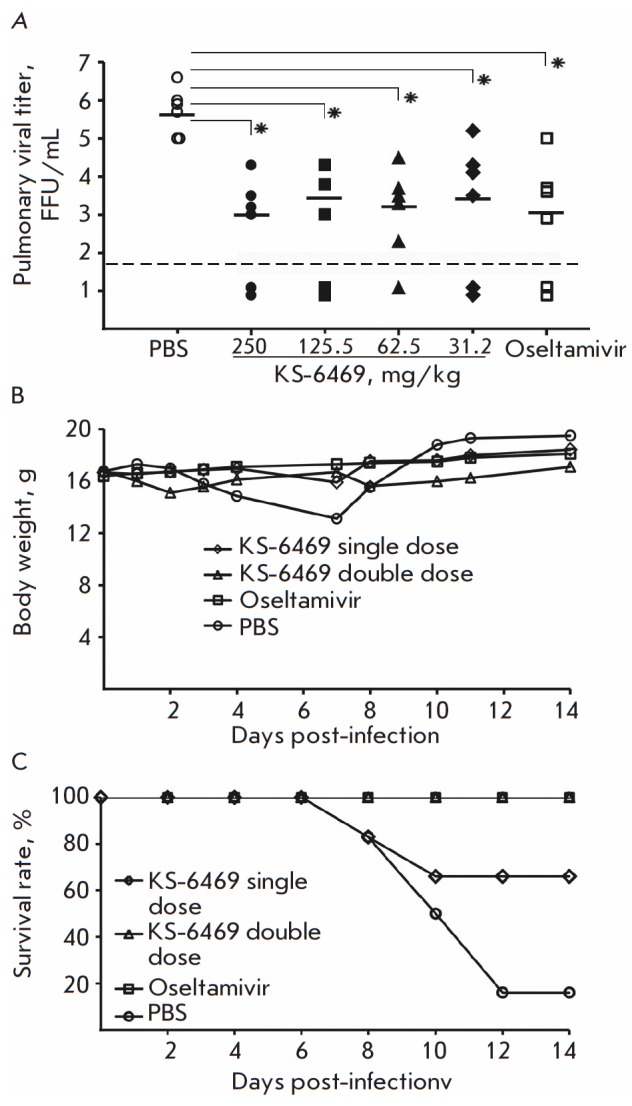
Protective efficacy of KS-6469 in a lethal challenge of IAV: A – BALB/c mice (n = 6/group) were treated intranasally with KS-6469 at the indicated doses and infected with 3 LD_50_ of IAV. The following day, the mice received a second dose of KS-6469 intranasally. The data show the pulmonary viral titers on day 3 p.i. by FFA. B – The average body weight of mice treated intranasally with KS-6469 at the indicated doses, oseltamivir (7.5 mg/kg) or PBS and infected with 3 LD_50_ of IAV. C – The survival rate of BALB/c mice (n = 6) treated intranasally with KS-6469 at the indicated doses, oseltamivir (7.5 mg/kg) or PBS and infected with 3 LD_50_ of IAV. Significance: *p < 0.05 vs. control group (without treatment)

Treatment of mice with KS-6469 confers protection against a lethal challenge of the influenza virus. The efficacy of KS-6469 against a lethal challenge of IAV was tested in the mouse model of influenza. To assess whether the compound could inhibit viral replication in vivo, we estimated the pulmonary viral titer in animals challenged with a lethal dose of 3 LD_50_ of IAV after the administration of different doses of KS-6469 via intranasal instillation. On day 2 p.i., the mice received a second dose of KS-6469 intranasally. The study included a positive control group that received oseltamivir daily (7.5 mg/kg), starting 30 min prior to viral challenge. On day 3 p.i., six animals per group were sacrificed and lung tissue extracts were analyzed by FFA (Fig. 8A). Viral titers were significantly reduced in the lungs of the animals treated with KS-6469 compared with those in the untreated group (p < 0.05), suggesting that intranasal therapy with KS-6469 inhibits influenza replication in mouse lungs (Fig. 8A). On day 3 p.i., the mock-treated mice had ~2.5 lg higher viral lung titers than the KS-6469- and oseltamivir-treated mice (p < 0.05). We suppose that the dose-independent antiviral activity of KS- 6469 may be related to the mechanism of action of the compound.

To compare the protective effect of KS-6469, two groups of mice were treated intranasally with KS- 6469 at a dose of 250 mg/kg, followed by challenge with a lethal dose of 3 LD_50_ IAV. On day 2 p.i., one of the two groups received a second dose of KS- 6469 intranasally. Mice in the positive control group were treated orally with oseltamivir at a dose of 7.5 mg/kg. The control animals were treated intranasally with PBS on the same schedule. The daily weight loss and survival rate were evaluated within 14 days. Figure 8B, C shows that two doses of KS- 6469 or treatment with oseltamivir led to minimal body weight loss with a 100% survival rate, which is significantly different than the untreated group (20% survival rate). The survival rate among mice treated with KS-6469 only once was 66%, and weight loss was minimal. Taken together, these data show that KS-6469 protects mice against a lethal infection of the influenza virus.

## DISCUSSION

Influenza remains one of the most dangerous viral diseases in existence. To date, only two classes of drugs have been approved for the treatment of influenza: M2 ion channel blockers (adamantanes) and NA inhibitors [23, 24]. Vaccination is a reliable way to control an influenza infection [25], but the emergence of mutated viruses has resulted in a low effectiveness of the influenza vaccine and antiviral therapeutic agents. Thus, there is an urgent need to develop new approaches to combat influenza. One of the possible solutions is to develop antiviral compounds based on sulfo derivatives of oligo- and polysaccharides derived from either synthetic or natural products. Although the antiviral studies are generally performed with enveloped viruses that are more susceptible to these compounds [3-7]; sulfated polysaccharides from marine microalgae have been shown to be active against non-enveloped viruses [26]. It is believed that sulfated polysaccharides target certain stages of the influenza replication cycle, tion. We hypothesize that viral inactivation occurs due to the interaction between KS-6469 and viral particles with the concomitant formation of aggregates, leading to a significant reduction in infectivity. Previously, it was suggested [21] that the virucidal activity of antiviral drugs observed in vitro would produce much more pronounced therapeutic effects in vivo. This assumption has been confirmed by the data presented in this study. We have demonstrated the antiviral efficacy of KS-6469 over a wide dose range in mice infected with IAV (Fig. 8). Intranasal treatment of influenza-infected mice with KS-6469 reduced the mortality rate in mice. Single application of KS-6469 increased the survival rate to 66%, while double application of the compound provided complete protection to the animals. The placebo-treated group showed only a 16% survival rate. Intranasal treatment of influenza-infected mice with KS-6469 markedly decreased the pulmonary viral titer even at the lowest dose used (62.5 mg/kg (Fig. 8)). such as binding of virus particles to the cell surface receptors of the host [27], internalization [28], mRNA and protein expression, and viral release [12, 29]. However, due to the manifold activities of sulfated polysaccharides, additional studies are required to elucidate the specific molecular mechanisms of their antiviral action. In the present study, we investigated the antiviral properties of a novel sulfonated derivative of β-cyclodextrin: KS-6469. We found that viral replication was completely suppressed after incubation of infected cells with 600 μg/mL of KS- 6469 for 24 h, indicating that the compound exhibits a high antiviral potential. To elucidate the mechanism of antiviral activity of KS-6469, we evaluated the effectiveness of inhibition at different stages of the virus replicative cycle (Fig. 3). Co-incubation of MDSK cells with KS-6469 did not affect the early stages of viral replication; however, the viral titer was significantly reduced when infected cells were co-incubated with KS-6469 for a long period of time. Based on these data, we hypothesized that KS-6469 affects the late stages of the influenza infection. The inhibitory effect of KS-6469 in the late stages of the infection might be related to the suppression of NA activity. We did not detect any changes in NA activity after incubation with KS-6469 (Fig. 5), but the activity of another viral envelope protein, hemagglutinin, decreased significantly under these conditions. After incubation of IAV with KS-6469 for 1 h at 37°C, the HA decreased fourfold compared with that in the untreated control, which did not lead to inhibition of the binding of viral hemagglutinin to sialic acid-containing receptors on cell surfaces (Table 1) and did not reduce virus reproduction. It should be noted that treatment with KS-6469 did not decrease the ability of viral hemagglutinin to interact with specific monoclonal antibodies (mAbs), suggesting that the structure of the hemagglutinin (the mAb target) remained intact (Fig. 4). Our results revealed that KS-6469 has pronounced virucidal activity, being able to cause complete loss of viral infectivity (Table 2). The virucidal activity of the compound was at its highest at 37°C but required a longer incubation period, which in turn explains the absence of KS-6469-induced inhibition during the early stages of viral replication. Treatment of the virus with KS- 6469 during the first 6 hours p.i. resulted only in a partial loss of infectivity and did not inactivate a sufficient number of virus particles to prevent infec

## CONCLUSIONS

Our study has shown that KS-6469 is a low-toxicity and safe agent that effectively inhibits the development of the infectious disease caused by the influenza virus. Intranasal application of KS-6469 had anti-IAV effects comparable to those of oseltamivir. Hence, KS- 6469 is a promising candidate for developing an effective antiviral drug for the prevention and treatment of influenza infections.

## Abbreviations

IAV: influenza A virus
PBS: phosphate-buffered saline
MDCK: Madin-Darby canine kidney cells
IMDM: Iscove’s Modified Dulbecco’s Medium
MOI: multiplicity of infection
IC50: compound concentration that produces 50% cell growth inhibition
CI: cell index; FFA – focus-forming assay
FFU: focus-forming unit
NA: neuraminidase
MUNANA: 2’-(4-methylumbelliferyl)-α-D-N-acetylneuraminic acid
HA: hemagglutination activity
p.i.: post-infection
MBCD: methyl-β-cyclodextrin
mAbs: monoclonal antibodies
